# DRG1 is a potential oncogene in lung adenocarcinoma and promotes tumor progression via spindle checkpoint signaling regulation

**DOI:** 10.18632/oncotarget.11973

**Published:** 2016-09-12

**Authors:** Li Lu, Yanrong Lv, Ji Dong, Shaohua Hu, Ruiyun Peng

**Affiliations:** ^1^ Department of Experimental Pathology, Beijing Institute of Radiation Medicine, Beijing, China; ^2^ Department of Breast Surgery, Qilu Hospital of Shandong University, Jinan, Shandong, China; ^3^ Hospital of PLA 96164 Troop, Jinhua, Zhejiang, China

**Keywords:** developmentally regulated GTP-binding protein 1, lung adenocarcinoma, spindle checkpoint, chemoresistance

## Abstract

Developmentally regulated GTP binding protein 1 (DRG1), a member of the DRG family, plays important roles in regulating cell growth. However, the molecular basis of DRG1 in cell proliferation regulation and the relationship between DRG1 and tumor progression remain poorly understood. Here, we demonstrate that DRG1 is elevated in lung adenocarcinomas while weakly expressed in adjacent lung tissues. DRG1 knockdown causes growth inhibition of tumor cells by significantly increasing the proportion of cells in M phase. Overexpression of DRG1 leads to chromosome missegregation which is an important index for tumorigenesis. Interestingly, ectopic of DRG1 reduces taxol induced apoptosis of lung adenocarcinoma cells. Mechanistic analyses confirm that DRG1 localizes at mitotic spindles in dividing cells and binds to spindle checkpoint signaling proteins *in vivo*. These studies highlight the expanding role of DRG1 in tumorigenesis and reveal a mechanism of DRG1 in taxol resistance.

## INTRODUCTION

Developmentally regulated GTP-binding protein (DRG) is a novel subfamily within the superfamily of GTP-binding protein, which displays little similarity with the well-characterized G-proteins except harboring the five characteristic motifs, G1–G5, that are believed to interact with GTP [1]. There are at least two distinct members, DRG1 and DRG2, from yeast to plant to human [2]. The two genes share 62% sequence identity at the nucleotide level and 58% identity at the protein level. The similar mRNA expression pattern in different tissues suggests that these two DRGs may have similar functions [2, 3]. Moreover, DRG1 and DRG2 can be regulated differently despite their structural similarities. DFRP1 specifically stabilizes DRG1 and Drg1/Dfrp1 complex could modulate protein synthesis mechanism in mammalian cells, whereas DFRP2 preferentially binds to DRG2 [4, 5].

Several studies have reported the correlation between DRG1 expression and proliferation. DRG1 is upregulated during the embryonic development of murine and downregulated after birth [6]. DRG1 shows increasing expression with feeding in *Atlantic salmon* and highest expression during the proliferative phase of the culture *in vitro* [7]. In plants, DRG1 is highly expressed in actively growing tissues and reproductive organs [3]. The expression patterns suggest that DRG1 plays important roles in proliferating cells. Since abnormal cellular proliferation is a characteristic of cancer cells, ectopic expression of DRG1 may contribute to the dysregulation of this normal control mechanism and induce tumorigenesis. In addition, ectopic expression of mouse DRG1 together with c-myc and ras stimulates cell transformation in fibroblast [8]. Studies also suggest that DRG1 is associated with SCL/TAL1 *in vivo* and *in vitro*, which is an oncogenic transcription factor for hematopoietic development [8, 9]. DRG1 from *Candida albicans* can contribute to the control of invasive filamentation and DRG1 deletion delays the invasive disease in the host [10]. Among all tumors, DRG1 has been proposed as a oncogene in melanoma, a marker for CPT-11 resistance in human head and neck xenograft tumors, and related gene in the recurrence probability of colon cancer [11–13]. However, little is known about the molecular mechanism underling growth regulation and it is unknown whether DRG1 plays a role in lung cancer, which is the leading cause of death from cancer [14].

In the present study, we identified DRG1 as a new oncogene in lung adenocarcinoma. We showed that loss of DRG1 induced mitosis arrest and growth inhibition in tumor cells. However, high DRG1 expression triggered abnormal chromosome segregation and decreased sensitivity to taxol-induced apoptosis. Specially, we reported that DRG1 acted as a novel mitotic spindle protein and binded to other spindle checkpoint signaling proteins which were the molecular basis for DRG1 inducing tumorigenesis and taxol resistance.

## RESULTS

### DRG1 is elevated in lung adenocarcinoma

**Figure 1:**
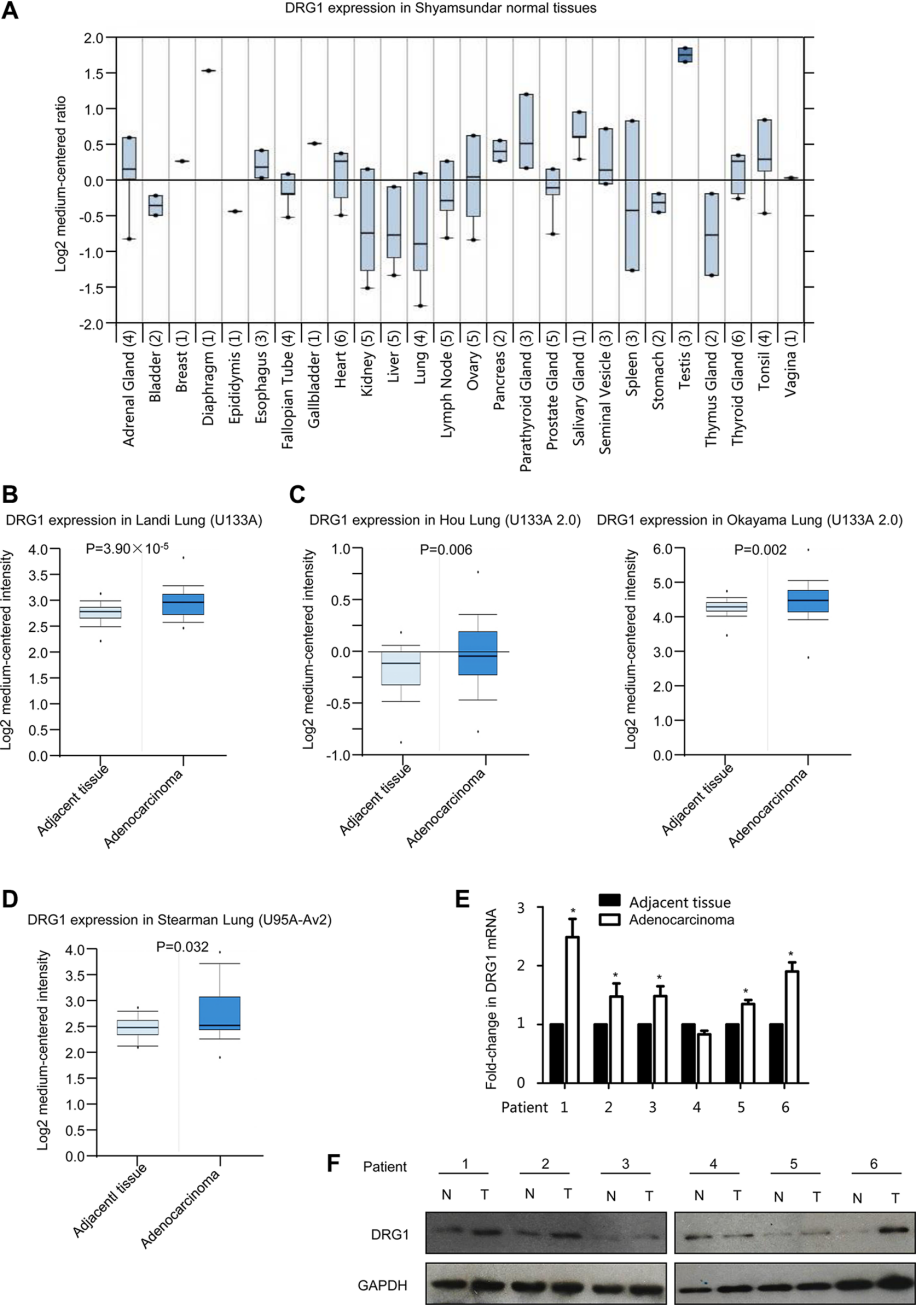
DRG1 is up-regulated in human lung adenocarcinoma (**A**) Box plot of DRG1 expression in twenty six kinds of tissues from oncomine. (**B**) Boxplots of DRG1 expression levels in lung adenocarcinoma and normal tissue samples using U133A microarray from oncomine. (**C**) Boxplots of DRG1 expression levels in lung adenocarcinoma and normal tissue samples using U133A 2.0 microarray from oncomine. (**D**) Boxplots of DRG1 expression levels in lung adenocarcinoma and normal tissue samples using U95A-Av2 microarray from oncomine. (**E**) The mRNA expression of DRG1 was performed by qRT-PCR in a subset of lung tumor tissues and matched adjacent normal control (T: tumor tissues; N: adjacent noncancerous tissues). Data were analyzed using Student's *t*-test. Data are shown as mean ± SEM of three independent experiments. *p < 0.05. (**F**) The protein expression of DRG1 was tested by western blot in a subset of lung tumor tissues and matched adjacent normal control (T: tumor tissues; N: adjacent noncancerous tissues).

To identify the expression pattern of DRG1 in tumors, we first performed a screen in oncomine database. The expression spectrum of DRG1 in various tissues suggested a lowest expression of DGR1 mRNA in normal lung tissues (Figure 1A), which was consistent with the previous report that DRG1 mRNA expressed weakly in both human and mouse lung tissues [2, 15]. Then, we confirm that DRG1 mRNA was significantly up-regulated in lung adenocarcinoma compared with adjacent tissues according to the microarray data in oncomine, including human genome U133A array data, U133 Plus 2.0 array data and U95A-Av2 array data (Figure 1B, 1C, 1D).

We then investigated whether there was evidence linking DRG1 protein to lung adenocarcinoma. DRG1 mRNA expression exhibited significant upregulation in randomly selected 5 out of 6 lung adenocarcinoma tissues (Figure 1E). Consistent with the results of DRG1 mRNA expression, DRG1 protein level was also highly up-regulated in adenocarcinoma tumor tissues compared with the adjacent tissues in 5 out of 6 patients (Figure 1F). Notably, expression levels of DRG1 were substantially increased in lung adenocarcinoma.

### Inhibition of DRG1 elicits a tumor suppressor effect by regulating cell cycle in lung cancer cells

**Figure 2:**
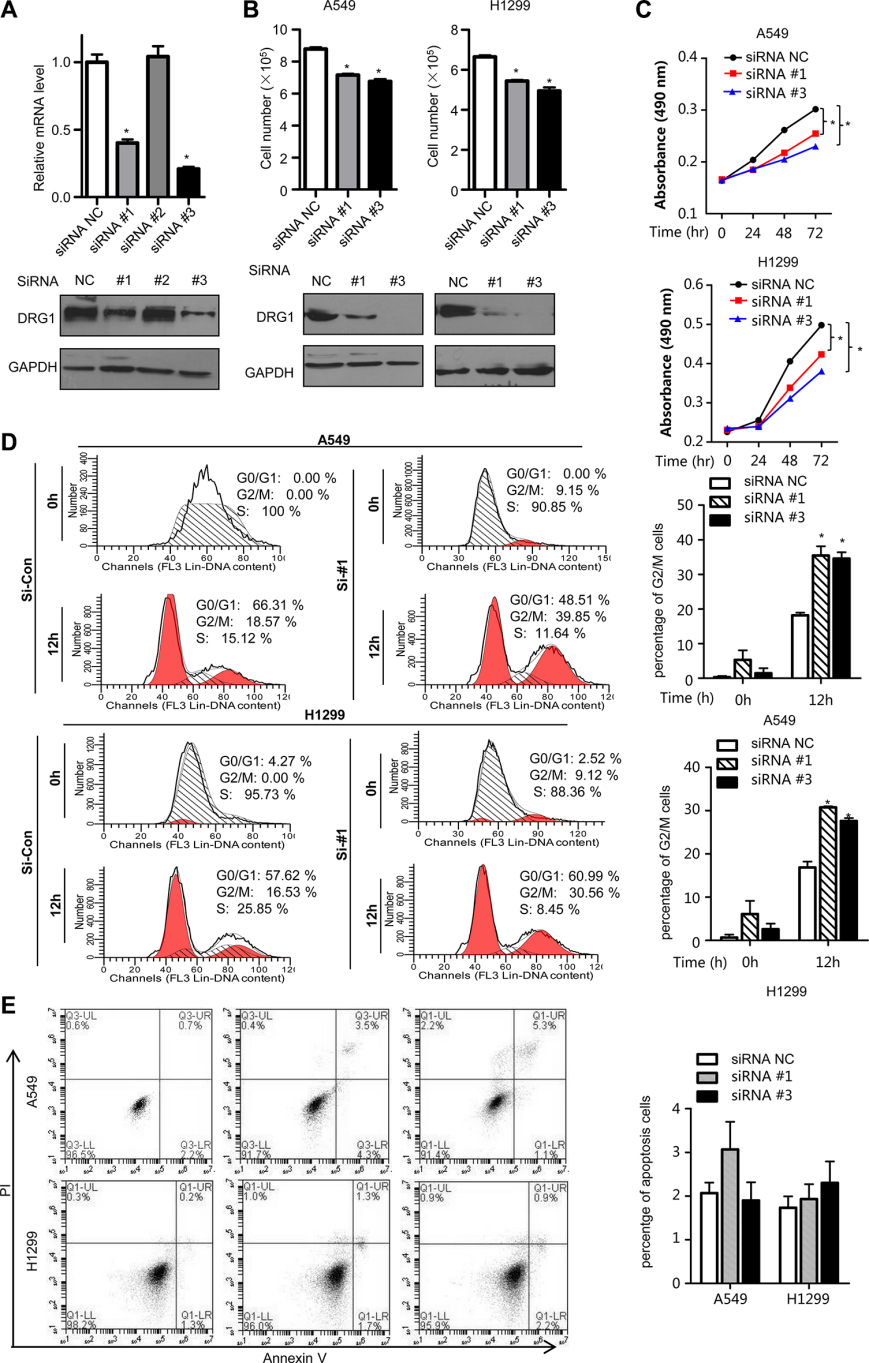
Effect of DRG1 knockdown on cell proliferation and cell cycle (**A**) DRG1 siRNA knockdown efficiency in A549 was measured by qRT-PCR and western blot. (**B**) H1299 and A549 cell lines were transfected with negative control siRNA or DRG1 specific siRNA. The cell numbers were determined using trypan blue cell counting after 72 hours. Data were analyzed using one-way ANOVA followed by Dunnett's post hoc test. Data are shown as mean ± SEM of three independent experiments. (**C**) A549 and H1299 cell lines were co-transfected with negative control siRNA or DRG1 specific siRNA. Cell proliferation was measured by MTS assay at the indicated time. Data were analyzed using two-way ANOVA. Data are shown as mean ± SEM of three independent experiments. (**D**) A549 and H1299 cells depleting DRG1 were synchronized at S phase by double-thymidine block. At the indicated hours after release from the block, cells lysates were collected and analyzed for DNA content by flow cytometry. *p < 0.05. (**E**) A549 and H1299 cells were transfected with control siRNA or DRG1 specific siRNA. Cells were stained with Annexin V and propidium iodide (PI) for analysis by flow cytometry.

To further understand the molecular functions of DRG1 in lung tumorigenesis, we performed small interfering RNA (siRNA)-mediated knockdown in A549 to test the efficiency of DRG1 specific siRNA. Two siRNAs were chosen for the following experiments (Figure 2A). Using the two DRG1 specific siRNA, we confirmed that DRG1 knockdown significantly reduced the growth rates and suppressed cell proliferation of A549 and H1299 (Figure 2B and 2C).

As it has been found that deregulation of cell cycle led to growth inhibition, we examined the effects of siRNA-mediated DRG1 loss on cell cycle. In A549, flow cytometry demonstrated that about 18% control cells were arrested with 4N DNA content 12h after release from the thymidine block, whereas about 35% DRG1 depleted cells had 4N DNA. The same result was shown in H1299 (Figure 2D). However, there was no significant difference in the proportion of apoptosis cells in the DRG1 knocking down A549 and H1299 cell lines (Figure 2E). This indicated that the predominant impact of DRG1 was to regulate cell cycle rather than cell apoptosis. In a word, DGR1 silencing inhibited mitotic progression but was not enough to cause cell death. M phase arrest might be one key mechanism by which DRG1 knockdown decreased lung tumor cell growth.

### Identification of DRG1 as a mitosis associated protein

All these previous results led us to explore the role of DRG1 in cell cycle regulation. To address this issue, we performed DAVID Gene Functional Classification on genes positively correlated (Pearson correlation > 0.6) with DRG1, and the co-expression data were based on primary tumor data using RNA-Seq [16]. Among all DRG1 positively correlated genes, most of them were involved in mitotic regulation. The proteins mainly participated in kinetochore complex and spindle assembly checkpoint (SAC) formation, such as CENPW, CENPN, CENPR, SPC25, OIP5, Bod1 and Mad2 (Table 1). Kinetochore complex regulates microtubule dynamics and is also required to generate spindle checkpoint signals in human cells. So the correlated genes of DRG1 in tumors suggested that it played a role in spindle checkpoint signals.

**Table 1:** The list of mitotic proteins co-expression with DRG1

Protein	Description	Other Aliases
BOD1	biorientation of chromosomes in cell division 1	FAM44B
CENPW	centromere protein W	CUG2
CENPN	centromere protein N	CENP-N
CCNB2	cyclin B2	HsT17299
CDKN3	cyclin-dependent kinase inhibitor 3	KAP
CKS1B	CDC28 protein kinase regulatory subunit 1B	CKS1
CKS2	CDC28 protein kinase regulatory subunit 2	CKSHS2
ERH	enhancer of rudimentary homolog (Drosophila)	DROER
GMNN	geminin, DNA replication inhibitor	Gem
HAUS1	HAUS augmin-like complex, subunit 1	CCDC5
ITGB3BP	integrin beta 3 binding protein (beta3-endonexin)	CENPR
MAD2L1	MAD2 mitotic arrest deficient-like 1 (yeast)	MAD2
MAD2L2	MAD2 mitotic arrest deficient-like 2 (yeast)	MAD2B
MITD1	microtubule interacting and trafficking domain containing 1	_
NUP37	nucleoporin 37 kDa	p37
RAN	RAN, member RAS oncogene family	Gsp1
SPC25	SPC25, NDC80 kinetochore complex component, homolog (S. cerevisiae)	SPBC25
OIP5	Opa interacting protein 5	MIS18B

The DRG1 positively correlated genes in primary tumors from TCGA were analyzed by gene functional classification from DAVID. Cell cycle related genes were listed in the table.

**Figure 3:**
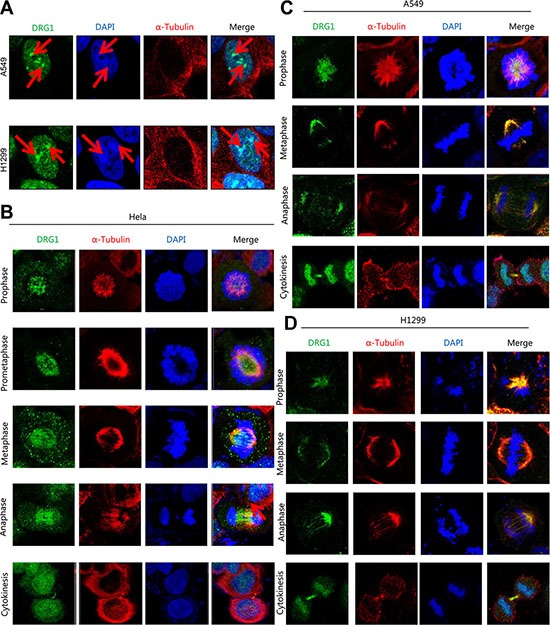
DRG1 is a spindle-associated protein (**A**) Non-dividing cells of H1299 and A549 were stained with 4, 6-diamidino-2-phenylindole (DAPI; for DNA; blue), anti-α-tubulin (microtubules; red) and anti-DRG1 (green). (**B**–**D**) The mitotic cells of HeLa, A549 and H1299 were separately stained with DAPI, anti-α-tubulin and anti-DRG1.

**Figure 4:**
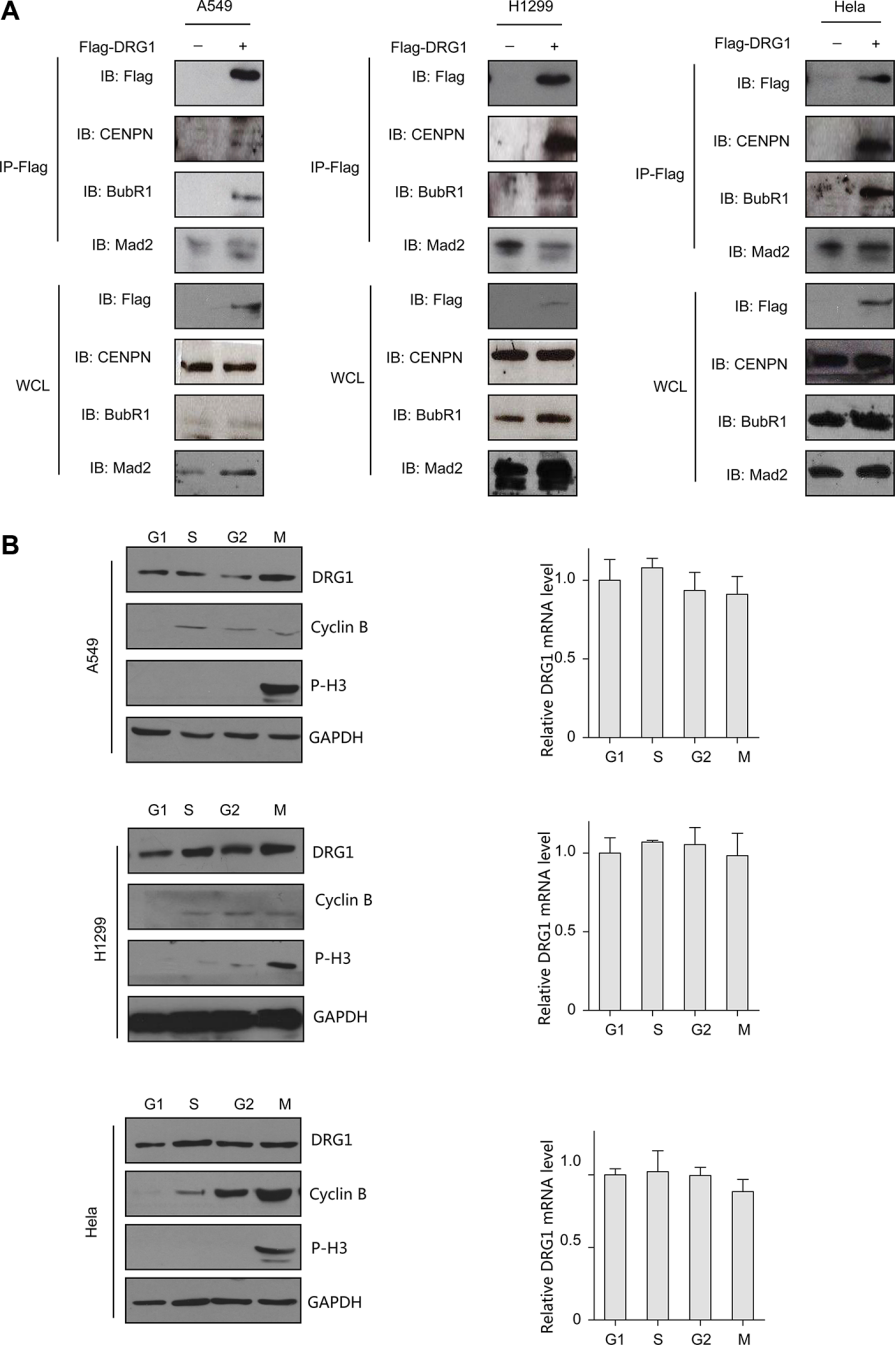
DRG1 interacts with mitotic proteins *in vivo* (**A**) Co-immunoprecipitation of Flag-DRG1 and endogenous mitotic protein is shown. A549, H1299, and HeLa cell were transfected with vector or Flag-DRG1. After 48 h, cell lysates were prepared and subjected to immunoprecipitation with Flag antibody and analyzed by immunoblotting using Flag or candidate protein antibodies. (**B**) Cells were synchronized at the indicated phase of cell-cycle, cells lysates were collected and analyzed by immunoblotting and Real-time RT-PCR for the proteins and mRNA level detection.

Given most of the cell cycle regulation proteins localized specially. DRG1 was observed throughout the nucleus and frequently observed in the perinucleolar boundary in non-dividing A549 and H1299 cells (Figure 3A), which indicated that DRG1 associated with the perinucleolar heterochromatin rings. However, DRG1 mainly localized at mitotic spindles during mitosis and colocalized with α-tubulin in HeLa, which was a cell line highly frequency used in cell cycle regulation analysis, A549 and H1299 (Figure 3B, 3C, 3D). Using primary antibody immunoprecipitation we confirmed the interaction of DRG1 with its co-expressing proteins in tumor cell lines. Endogenous Mad2 and BubR1, two SAC proteins, were co-immunoprecipitated with Flag-DRG1 in A549, H1299 and HeLa cells (Figure 4A). Additionally, Flag-DRG1 could also bind to the endogenous CENPN, which was a kinetochore protein. In a word, DRG1 was a novel spindle protein, and it functioned together with the proteins involved in spindle checkpoint signaling. All these data supported our hypothesis that DRG1 was involved in the organization and regulation of mitotic spindle checkpoint.

We next determined the expression level of DRG1 in G1, S, G2, and M phase. Thymidine-nocodazole arrested cells were mitotic cells. For G1 phase cells, M phase arrested cells were shaken off and released into fresh medium for 4 hours. For S phase and G2 phase cells, A549, H1299 and HeLa cells were synchronized by a double-thymidine protocol, and collected after releasing arrested cells into fresh medium for 4 and 8 hours. Western blot analysis and qPCR showed that the protein level and mRNA level of DRG1 was similar across the cell cycle (Figure 4B), which was different from the other proteins dynamic changing during the whole cell cycle. So DRG1 might have other functions besides cell cycle regulation.

### Overexpression of DRG1 induces abnormal chromosomal segregation

**Figure 5:**
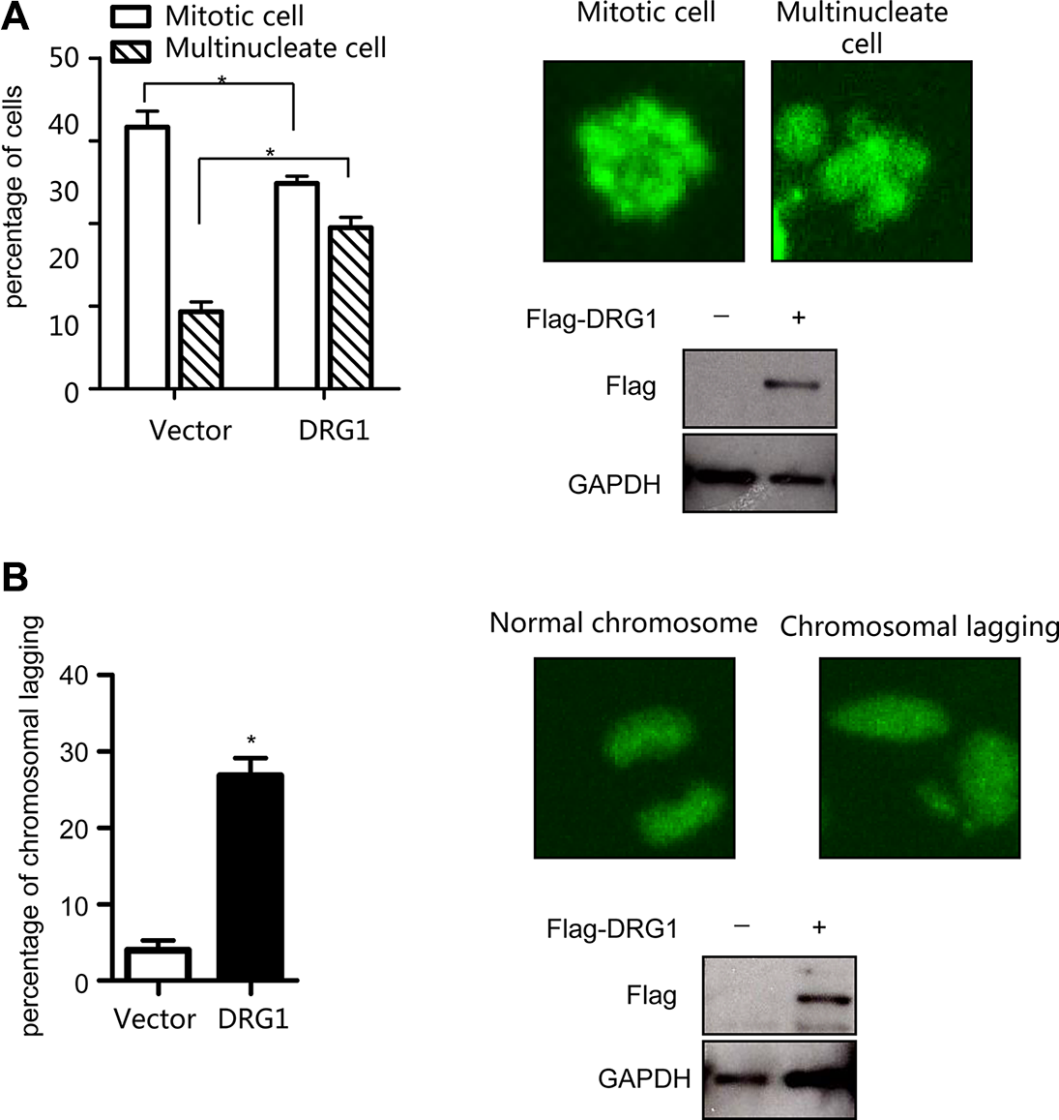
Overexpression of DRG1 leads to chromosome missegregation (**A**) HeLa/GFP-H2B cells overexpressed with DRG1 were synchronized by double-thymidine block and treated with taxol (100 ng/ml). The mitotic index and multilobed nucleus index were determined. Data were analyzed using Student's *t*-test. Data are represented as mean ± SEM of three independent experiments. Representative images and DRG1 expression level are shown on the right. (**B**) HeLa/GFP-H2B cells expressing DRG1 were detected normal and lagging chromosomes. Data were analyzed using Student's *t*-test. Data are represented as mean ± SEM of three independent experiments. Representative images and DRG1 expression level in HeLa cells are shown on the right. *p < 0.05.

To confirm our findings that DRG1 functioned together with SAC and kinetochores proteins, we investigated the effect of DRG1 on spindle checkpoint signaling. Using the HeLa cell line stably co-expressed a histone H2B-GFP, we tested whether overexpression of DRG1 could override taxol-induced mitotic arrest. Cells transfected with Flag-DRG1 were first synchronized by double-thymidine block, and then cultured with medium containing Taxol. When the cells began to go into M phase, observed the cells by time-lapse. The percentage of cells with multilobed nuclei was much higher in DRG1-expressing cells than in vector-expressing cells after 24 hours treatment (Figure 5A). Moreover, the number of cells with lagging chromosomes increased in DRG1-expressing cells (Figure 5B). These results indicated that overexpression of DRG1 led to the unstability of microtubule and dysregulated the spindle checkpoint signaling, which could cause chromosome instability and promote tumorigenesis in certain contexts. Obviously, DRG1 participated in the regulation of mitosis, and this was one of the mechanisms that overexpression of DRG1 triggered lung adenocarcinoma.

### Overexpression of DRG1 decreases sensitivity to chemotherapy-induced apoptosis

**Figure 6:**
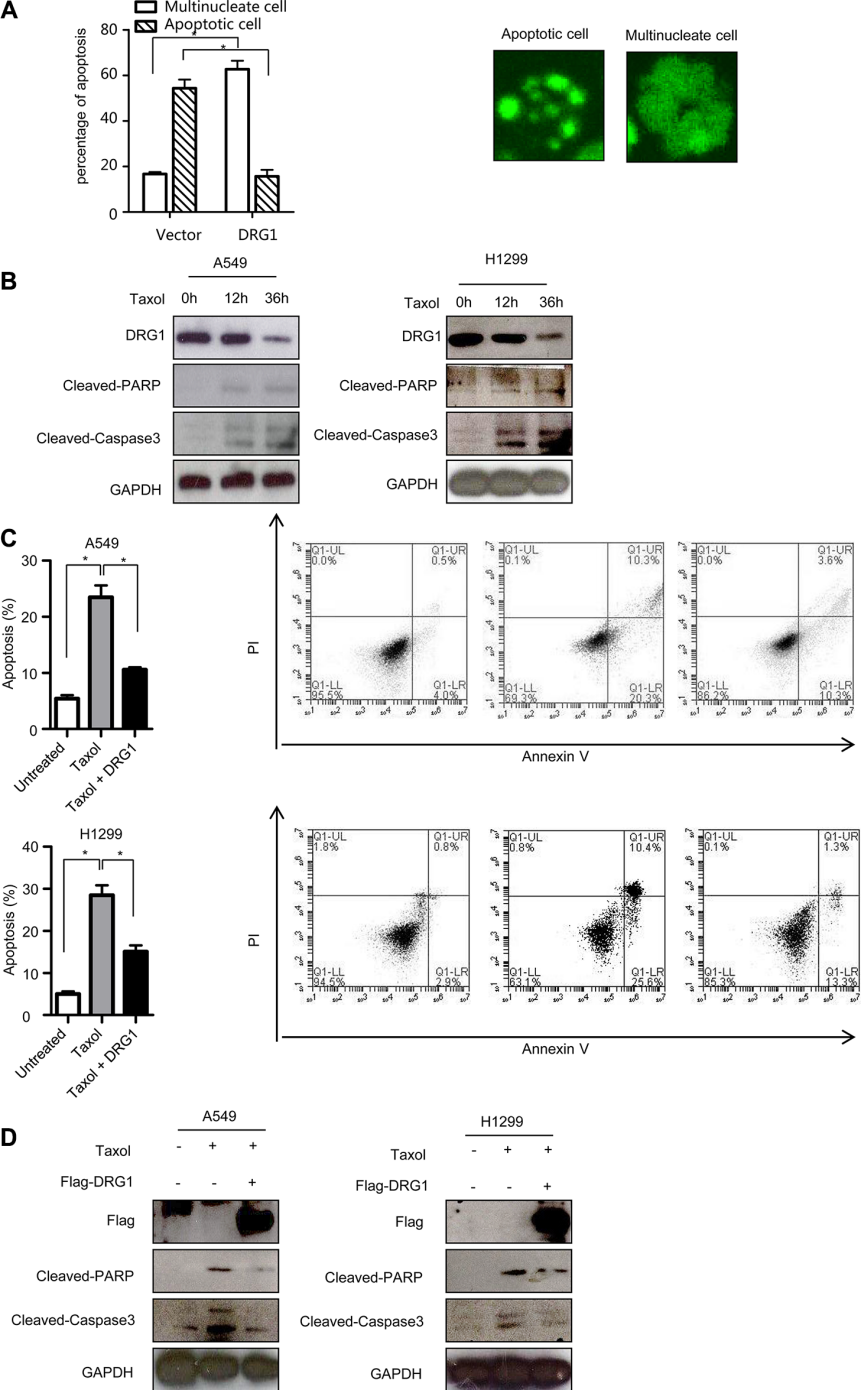
Ectopic of DRG1 decreases the apoptosis induced by taxol (**A**) HeLa/GFP-H2B cells overexpressed with DRG1 were synchronized by double-thymidine block and treated with taxol (100 ng/ml). After 36 h, the apoptosis index and multilobed nucleus index were determined. Data were analyzed using Student's *t*-test. Data are represented as mean ± SEM of three independent experiments. Representative images are shown on the right. (**B**) A549 and H1299 cells were treated with taxol (100 ng/ml) for indicated times and the indicated proteins were detected by western blot. (**C**) A549 and H1299 cells were transfected with vector or Flag-DRG1, and then cells were treated with taxol (100 ng/ml). Cells were stained with Annexin V and propidium iodide (PI) for analysis by flow cytometry after 36 h treatment. (**D**) A549 and H1299 cells transfected with or without Flag-DRG1 were either untreated or treated with taxol (100 ng/ml) for 36 h, and then the indicated proteins were examined by western blot. *p < 0.05.

Overexpression of DRG2, another family member of DRGs, decreases sensitivity to nocodazole-induced apoptosis in Jurkat cells [17]. To fully characterize the oncogenic effects of DRG1 overexpression, we evaluated whether DRG1 influence the chemotherapy response induced by taxol. We extended the time-lapse hours for observing the DRG1-expressing cells treated with taxol. Many of cells-expressing control were apoptotic after 36 hours, but more cells-expressing DGR1 were also alive in a multilobed nuclei manner (Figure 6A). So DRG1 contributed to the resistance of taxol induced apoptosis.

We checked the endogenous protein level of DRG1 during chemotherapy-induced apoptosis. Upon the taxol treatment, the active fragment of caspase 3 and cleavage of PARP, two apoptosis markers, were induced in A549 and H1299. However, DRG1 protein was significantly decreased after taxol treatment (Figure 6B). Otherwise, DRG1 overexpression significantly suppressed taxol induced cell death compared with control (Figure 6C) and reduced the protein levels of the cleaved caspase 3 and PARP at the same time (Figure 6D). Overall, level of DRG1 expression could help to predict the sensitivity of cancer cells to taxol.

## DISCUSSION

Adenocarcinoma is the most common form of lung cancer, which is the leading cause of cancer death [14, 18]. Here, we identified that DRG1 was involved in spindle checkpoint signaling, and provided new avenue for tumorigensis and potential important predictive marker of taxol resistance. As a homologous, DRG2 was also reported as a chemotherapeutic drugs resistance marker in hepatocellular carcinoma cells [19]. However, downregulation of DRG2 could prevent the apoptosis of lung cancer cells. Absolutely, the functional differences between DRG1 and DRG2 have been independently argued since the two proteins were first recognized.

DRG1 was demonstrated to localize in nuclear and especially on mitotic spindles through mitosis. Previous study showed different localization pattern of DRG1. DRG1 protein was detected in the perinuclear and mainly in cytoplasm in murine and human at earlier [15, 20, 21]. Several reports pointed that the interacting protein of DRG1 were localized in the nuclear, such as UBC9, SCL/TAL1 [9, 22]. As a DRG1-associated protein, p48ZnF localized at nuclear in the proliferating cells and at cytoplasmic in the differentiated neuron [23]. DRG1 cellular location may also depend on the cell's differentiation status like p48ZnF.

To our knowledge, this is the first report to reveal the potential interaction of DRG1 with cell cycle proteins. Kinetochore protein (CENPN) and spindle assembly checkpoint proteins (Mad2 and BubR1) were confirmed as the interaction protein of DRG1 *in vivo*. CENPN is required to recruit the Mad2 SAC protein to prometaphase kinetochores [24]. MAD2 is a key component of the mitotic spindle checkpoint pathway, and MAD2 overexpression is observed in several cancers [25]. MAD2 overexpression in transgenic mice notably results in chromosomal instability and initiates carcinogenesis in a wide variety of tumors [26]. We believed that DRG1 function together with these proteins and play important roles in spindle checkpoint signaling, because DRG1 overexpression resulted in chromosomal instability like Mad2 and showed taxol resistance. Of course, further experimentations will be needed to support the conclusion and other high-correlation proteins are worth to be further studied.

DRG1 participated in the regulation of chromosome segregation, but we do not know the deeper mechanism that how DRG1 function. SUMOylation is a protein conjugation process which leads to covalent modification of many proteins involved in chromosome segregation [27]. DRG1 is the SUMOylation substrate of UBC9 [22]. Otherwise, mimicking phosphorylation of Thr100 decreases DRG1 activity [28]. DRG1 is subjected to a tight regulation, and it will be interesting to investigate whether SUMOylation, phosphorylation and DRG1 GTPase activity is critical for its mitosis regulation.

In conclusion, we uncovered the upregulated expression of DRG1 in lung adenocarcinoma. This was the first time to show that DRG1 was an important protein involved in spindle checkpoint signaling, and elevated levels of DRG1 caused lung adenocarcinoma and taxol resistance. It will be worth to further investigate the relationship between the imbalance of DRG1 in mitotic progression and tumorigenesis.

## MATERIALS AND METHODS

### Cell culture and transfection

A549 cells, H1299cells, HeLa cells, and HeLa/GFP-H2B cells were cultured in RPMI1640 supplemented with 10% fetal bovine serum (FBS) and 1% antibiotic-antimycotic. All cell lines were cultured at 37°C in a 5% CO_2_ cell culture incubator. Cells were transfected with Lipofectamine 2000 following the manufacturer's protocol (Invitrogen).

### Antibody and reagents

Nocodazole, thymidine, taxol and antibody DRG1, CENPN, and Flag were purchased from Sigma-Aldrich. Anti-GAPDH and secondary antibodies were purchased from Santa Cruz Biotechnology. Antibody against α-tubulin, cyclinB, P-H3, Mad2, and BubR1 were purchased from Abcam. Antibody against cleaved-caspase3 and cleaved-PARP were purchased from Cell Signaling Technology.

### Tissue samples and Real-time RT-PCR

Lung adenocarcinoma and matched adjacent normal lung tissue samples used in this analysis were obtained during surgical removal of tumors from patients histopathologically diagnosed with lung adenocarcinoma in QiLu Hospital of Shandong University. The protocol was approved by the Hospital's Protection of Human Subjects Committee and written informed consent was obtained from all patients before sample collection. All samples were immediately snap-frozen in liquid nitrogen and stored at −80°C for analysis.

Total RNA was isolated from the cells or tissues using TRIzol (Invitrogen) and reversed-transcribed using 1 μg of total RNA with an oligo-dT primer. The following primers were used for real-time PCR: human GAPDH forward, 5′-GGGAAGGTGAAGGTCGGAGT-3′; GAPDH reverse, 5′-TTGAGGTCAATGAAGGGGTCA-3′; human DRG1 forward, 5′-TGGAGGTCCAGGAGAAGGTTT-3′; and DRG1 reverse, 5′-GCCACTGCAATGACTTGACG-3′.

### RNA interference

The DRG1 siRNA-no.1 (5′-GACCAUACGUU GGAGGAUGTT-3′), siRNA-no.2 (5′-GGUAGAGGUC GUCAAGUCATT-3′), siRNA-no.3 (5′-GGCCAGUU ACCAGAUUACATT-3′) and non-targeting siRNAs (5′-UUCUCCGAACGUGUCACGU-3′) were synthesized by Shanghai GenePharm. All siRNAs were transfected into the cells according to the manufacturer's protocol.

### Immunoprecipitation and immunoblotting

Total cell lysate were prepared in HEPES lysis buffer (20 mM HEPES pH 7.2, 50 mM NaCl, 0.5% Triton, X-100, 1 mM NaF, 1 mM dithiothreitol) and boiled with SDS/PAGE loading buffer. Proteins were separated by SDS-PAGE, transferred onto NC membrane and examined by immunoblotting with the indicated primary antibodies and appropriate secondary antibody, followed by detection with Super Signal chemiluminescence kit (Pierce). For immunoprecipitation, cells were lysed in HEPES lysis buffer supplemented with protease inhibitor cocktail (Roche). Immunoprecipitation was performed using indicated antibody for 4 hour at 4°C followed by incubation with protein A/G-agarose beads (Santa Cruz) overnight at 4°C. Beads were then washed three times in HEPES lysis buffer and examined by immunoblotting.

### Cell proliferation assay

3-(4, 5-dimethylthiazol-2-yl)-5-(3-carboxymethoxy- phenyl)-2-(4-sulfophenyl)-2H-tetrazolium (MTS) assays were performed to determine relative cell proliferation. Cells were plated in a 96-well plate and transfected with negative control siRNA or DRG1 siRNA. At 0 h, 24 h, 48 h, and 72 h after transfection, the cells were incubated in Cell Proliferation Reagent (Promega) according to manufacturer's instructions.

### Apoptosis assay

Cells were seeded in the 6-well plate, and cells were trypsinized and rinsed in PBS after treatment. Then, 5 ul of Annexin V-FITC (BD Biosciences, San Diego, CA, USA) and 5 ul of propidium iodide (PI) solution were added. Cells were incubated at room temperature for 15 min in dark. After incubation, a 400 ul 1 × binding buffer was put to each tube, and apoptotic fractions were detected using the flow cytometry.

### Immunofluorescence staining and time–lapse imaging

For subcellular localization analyses, cells were grown on glass chamber and fixed with 4% paraformaldehyde, permeabilized in 0.2% Triton X-100/PBS and blocked with 5% BSA. Proteins were stained using the indicated antibodies and detected with a TRITC-conjugated or FITC-conjugated secondary antibody. The nuclei were stained with DAPI (Sigma), and images were visualized with a Zeiss LSM 510 Meta inverted confocal microscope.

For time-lapse imaging, HeLa cell line stably co-expressing a histone H2B-GFP was used to visualize chromosomes. Cells were seeded in an eight-chambered cover glass (Lab-Tek Chambered no 1.0 Borosilicate Cover Glass System, Nunc). The temperature of the imaging medium was kept at 37°C. After treatment images were collected every 5 min using a 40 (or 20) lens objective on inverted fluorescence microscope (Nikon Eclipse Ti-E) with an Ultra View spinning-disc confocal scanner unit (Perkin Elmer). Image sequences were viewed using Volocity software, and cell behavior was analyzed manually.

### Cell cycle analysis by flow cytometry

The Cell synchronization was performed as previously described [29]. A549 and H1299 cells were collected and then fixed with 70% ice-cold ethanol after treatment, washed with PBS, re-suspended in 1 ml of propidium iodide (PI) staining solution (0.1% Triton X-100, 10 μg/mL PI, and 100 μg/mL DNase-free RNase A in PBS), and then incubated for 30 min at room temperature in the dark. Samples were transferred to the flow cytometer and the cell cycle distribution was analyzed using flow cytometry.

### Published RNA sequencing data collections

Oncomine database [30] were screened for mRNA expression of DRG1, four published microarray data sets of primary lung AD tissues were utilized. These included Hou et al., with 45 LUADs and 65 lungs [31], Landi et al., with58 LUADs and 49 lungs [32], Stearman et al., with 20 LUADs and 19 lungs [33], and Okayama et al., with 226 LUAD and 20 lungs [34]. DRG1 co-expression genes were collected in TCGA cohorts [16].

## References

[1] Schenker T, Lach C, Kessler B, Calderara S, Trueb B (1994). A novel GTP-binding protein which is selectively repressed in SV40 transformed fibroblasts. J Biol Chem.

[2] Li B, Trueb B (2000). DRG represents a family of two closely related GTP-binding proteins. Biochim Biophys Acta.

[3] Etheridge N, Trusov Y, Verbelen JP, Botella JR (1999). Characterization of ATDRG1, a member of a new class of GTP-binding proteins in plants. Plant Mol Biol.

[4] Ishikawa K, Azuma S, Ikawa S, Semba K, Inoue J (2005). Identification of DRG family regulatory proteins (DFRPs): specific regulation of DRG1 and DRG2. Genes Cells.

[5] Ishikawa K, Akiyama T, Ito K, Semba K, Inoue J (2009). Independent stabilizations of polysomal Drg1/Dfrp1 complex and non-polysomal Drg2/Dfrp2 complex in mammalian cells. Biochem Biophys Res Commun.

[6] Kumar S, Tomooka Y, Noda M (1992). Identification of a set of genes with developmentally down-regulated expression in the mouse brain. Biochem Biophys Res Commun.

[7] Bower NI, Johnston IA (2010). Discovery and characterization of nutritionally regulated genes associated with muscle growth in Atlantic salmon. Physiol Genomics.

[8] Mahajan MA, Park ST, Sun XH (1996). Association of a novel GTP binding protein, DRG, with TAL oncogenic proteins. Oncogene.

[9] Zhao XF, Aplan PD (1998). SCL binds the human homologue of DRG *in vivo*. Biochim Biophys Acta.

[10] Chen X, Kumamoto CA (2006). A conserved G protein (Drg1p) plays a role in regulation of invasive filamentation in Candida albicans. Microbiology.

[11] Kiniwa Y, Li J, Wang M, Sun C, Lee JE, Wang RF, Wang HY (2015). Identification of DRG-1 As a Melanoma-Associated Antigen Recognized by CD4+ Th1 Cells. PLoS One.

[12] Azrak RG, Yu J, Pendyala L, Smith PF, Cao S, Li X, Shannon WD, Durrani FA, McLeod HL, Rustum YM (2005). Irinotecan pharmacokinetic and pharmacogenomic alterations induced by methylselenocysteine in human head and neck xenograft tumors. Mol Cancer Ther.

[13] Sebio A, Gerger A, Matsusaka S, Yang D, Zhang W, Stremitzer S, Stintzing S, Sunakawa Y, Yamauchi S, Ning Y, Fujimoto Y, Ueno M, Lenz HJ (2015). Genetic variants within obesity-related genes are associated with tumor recurrence in patients with stages II/III colon cancer. Pharmacogenet Genomics.

[14] Siegel RL, Miller KD, Jemal A (2015). Cancer statistics, 2015. CA Cancer J Clin.

[15] Sazuka T, Kinoshita M, Tomooka Y, Ikawa Y, Noda M, Kumar S (1992). Expression of DRG during murine embryonic development. Biochem Biophys Res Commun.

[16] Cancer Genome Atlas Research N (2014). Comprehensive molecular profiling of lung adenocarcinoma. Nature.

[17] Song H, Kim SI, Ko MS, Kim HJ, Heo JC, Lee HJ, Lee HS, Han IS, Kwack K, Park JW (2004). Overexpression of DRG2 increases G2/M phase cells and decreases sensitivity to nocodazole-induced apoptosis. J Biochem.

[18] Matsuda T, Machii R (2015). Morphological distribution of lung cancer from Cancer Incidence in Five Continents Vol. X. Jpn J Clin Oncol.

[19] Chen J, Shen BY, Deng XX, Zhan Q, Peng CH (2012). SKP1-CULLIN1-F-box (SCF)-mediated DRG2 degradation facilitated chemotherapeutic drugs induced apoptosis in hepatocellular carcinoma cells. Biochem Biophys Res Commun.

[20] Sazuka T, Tomooka Y, Ikawa Y, Noda M, Kumar S (1992). DRG: a novel developmentally regulated GTP-binding protein. Biochem Biophys Res Commun.

[21] Sommer KA, Petersen G, Bautz EK (1994). The gene upstream of DmRP128 codes for a novel GTP-binding protein of Drosophila melanogaster. Mol Gen Genet.

[22] Jakobs A, Himstedt F, Funk M, Korn B, Gaestel M, Niedenthal R (2007). Ubc9 fusion-directed SUMOylation identifies constitutive and inducible SUMOylation. Nucleic Acids Res.

[23] Heese K (2013). Establishing an *in vivo* p48ZnF bioluminescence mouse brain imaging model. Neurosci Lett.

[24] Matson DR, Demirel PB, Stukenberg PT, Burke DJ (2012). A conserved role for COMA/CENP-H/I/N kinetochore proteins in the spindle checkpoint. Genes Dev.

[25] Nakano Y, Sumi T, Teramae M, Morishita M, Fukuda T, Terada H, Yoshida H, Matsumoto Y, Yasui T, Ishiko O (2012). Expression of the mitotic-arrest deficiency 2 is associated with chemotherapy resistance in ovarian serous adenocarcinoma. Oncol Rep.

[26] Sotillo R, Hernando E, Diaz-Rodriguez E, Teruya-Feldstein J, Cordon-Cardo C, Lowe SW, Benezra R (2007). Mad2 overexpression promotes aneuploidy and tumorigenesis in mice. Cancer Cell.

[27] Hay RT (2005). SUMO: a history of modification. Mol Cell.

[28] Perez-Arellano I, Spinola-Amilibia M, Bravo J (2013). Human Drg1 is a potassium-dependent GTPase enhanced by Lerepo4. FEBS J.

[29] Lu L, Hu S, Wei R, Qiu X, Lu K, Fu Y, Li H, Xing G, Li D, Peng R, He F, Zhang L (2013). The HECT type ubiquitin ligase NEDL2 is degraded by anaphase-promoting complex/cyclosome (APC/C)-Cdh1, and its tight regulation maintains the metaphase to anaphase transition. J Biol Chem.

[30] Rhodes DR, Yu J, Shanker K, Deshpande N, Varambally R, Ghosh D, Barrette T, Pandey A, Chinnaiyan AM (2004). ONCOMINE: a cancer microarray database and integrated data-mining platform. Neoplasia.

[31] Hou J, Aerts J, den Hamer B, van Ijcken W, den Bakker M, Riegman P, van der Leest C, van der Spek P, Foekens JA, Hoogsteden HC, Grosveld F, Philipsen S (2010). Gene expression-based classification of non-small cell lung carcinomas and survival prediction. PLoS One.

[32] Landi MT, Dracheva T, Rotunno M, Figueroa JD, Liu H, Dasgupta A, Mann FE, Fukuoka J, Hames M, Bergen AW, Murphy SE, Yang P, Pesatori AC (2008). Gene expression signature of cigarette smoking and its role in lung adenocarcinoma development and survival. PLoS One.

[33] Stearman RS, Dwyer-Nield L, Zerbe L, Blaine SA, Chan Z, Bunn PA, Johnson GL, Hirsch FR, Merrick DT, Franklin WA (2005). Analysis of orthologous gene expression between human pulmonary adenocarcinoma and a carcinogen-induced murine model. The American journal of pathology.

[34] Okayama H, Kohno T, Ishii Y, Shimada Y, Shiraishi K, Iwakawa R, Furuta K, Tsuta K, Shibata T, Yamamoto S (2012). Identification of genes upregulated in ALK-positive and EGFR/KRAS/ALK-negative lung adenocarcinomas. Cancer research.

